# Enhanced Numerical Equivalent Acoustic Material (eNEAM): Analytical and Numerical Framework for Porous Media with Thermo-Viscous Effects for Time Domain Simulations

**DOI:** 10.3390/ma18235441

**Published:** 2025-12-02

**Authors:** P. C. Iglesias, L. Godinho, J. Redondo

**Affiliations:** 1 Instituto de Investigación para la Gestión Integrada de Zonas Costeras, Universitat Politècnica de València, Campus de Gandía, C. Paranimf, 1., 46730 Gandia, Spain; fredondo@fis.upv.es; 2 Departamento de Engenharia Civil, Universidade de Coimbra, ISISE, ARISE, R. Luis Reis dos Santos 290, 3030-790 Coimbra, Portugal; lgodinho@dec.uc.pt

**Keywords:** FDTD acoustic modeling, porous absorbers, thermo-viscous dissipation, multi-scale simulations

## Abstract

Accurate prediction of sound propagation in porous and dissipative media remains challenging when classical models struggle to capture the microscopic material characteristics. This work introduces the Enhanced Numerical Equivalent Acoustic Material (eNEAM) framework, extending the original NEAM formulation by combining analytical and numerical approaches. The analytical formulation provides closed-form expressions for effective impedance, complex wavenumber, and absorption coefficient under normal incidence, with and without thermo-viscous effects, enabling a direct validation against impedance-tube data and efficient initialization of finite-difference time-domain (FDTD) simulations. A parameter optimization strategy, focused on the thermolabile coefficient ($\Psi_{B}$), significantly improves low-frequency absorption predictions. Robustness studies reveal that even substantial variations in model parameters generally remain within an optimal ±10% range. Additionally, a comparison between models with and without thermo-viscous losses was performed and shows that differences are negligible at macroscopic scales, which can be useful to reduce computational costs. Following computational time reduction, the adaptive mesh refinement technique employed also reduces time costs by over 50% in 1-D FDTD simulations, even without GPU acceleration. Taken together, these developments demonstrate that eNEAM provides a versatile, accurate, and computationally efficient framework for modeling porous materials, bridging experimental characterization, analytical formulations, and numerical simulations while maintaining robustness against parameter variations.

## 1. Introduction

Accurate prediction of sound propagation in dissipative materials is essential for applications ranging from architectural acoustics to noise control and metamaterial design. Classical empirical models, such as Delany–Bazley [1], or semi-phenomenological formulations like Johnson–Champoux–Allard–Lafarge (JCAL) [2,3,4], provide effective estimates of characteristic impedance and wavenumber under equilibrium assumptions. However, they are limited when frequency-dependent losses exceed the visco-inertial regime [5] or when non-equilibrium relaxation phenomena become significant [6,7].

Recent advances have addressed some of these limitations. For instance, Jaouen [8] proposed a complete estimation method of the six JCAL parameters from impedance-tube data, enabling more accurate identification of porous material properties. Similarly, Bouchendouka [9] extended formulations by introducing additional viscous and thermal parameters (Σ, V, Σ′, V′) and has been reported to model wave propagation in highly attenuating porous media up to ultrasonic frequencies. In addition, Bouchendouka [10] further refined viscous and thermal dynamic tortuosities for highly resistive porous materials through a combined theoretical and experimental approach. These works highlight that accurate representation of microstructural dissipation mechanisms is critical for modeling porous materials where conventional JCAL parameters are not sufficiently accurate. Beyond parameter estimation, theoretical work has also expanded the JCAL framework: Lafarge [11] introduced spatial dispersion into effective tortuosity and compressibility models, capturing non-local effects that dominate in micro-structured media. These developments are crucial for simulating complex absorbers, metamaterials, or heterogeneous configurations where boundary layers and multi-scale structures govern acoustic response. However, their main limitation is the requirement for highly accurate parameter characterization, which is difficult to achieve in practice due to measurement constraints and instrumentation limits, introducing unavoidable uncertainty into the results.

In parallel, numerical and experimental studies of topologically optimized porous metamaterials, such as triply periodic minimal surface (TPMS) lattices, have demonstrated that geometry, porosity, and cell size strongly control absorption, particularly in the 2–5 kHz range [12]. Advanced computational schemes based on the linearized Navier–Stokes equations have further clarified the regions of dominant thermo-viscous dissipation in porous microstructures, providing validation frameworks for reduced-order models [13]. Moreover, recent reviews highlight the importance of robust characterization methods, standardized procedures, and the inclusion of Willis cross-coupling in heterogeneous media to better capture non-equilibrium effects [14].

In this context, the Numerical Equivalent Acoustic Material (NEAM) concept [15] addresses the challenge by introducing numerical coefficients that reproduce the acoustic behavior of a porous material through equivalent macroscopic parameters, without requiring explicit microscopic characterization or other complexes approaches. The linearized NEAM framework uses four coefficients derived from impedance-tube measurements, enabling accurate reconstruction of material impedance and absorption of air-filled porous materials. However, small but systematic discrepancies were observed between the NEAM predictions and measured absorption, particularly in the low-frequency regime. In this work, it is demonstrated that an improved parameter search strategy, focused on optimizing the numerical thermolabile parameter ($\Psi_{B}$), allows the NEAM model to reproduce the experimental data with significantly higher fidelity.

Moreover, to establish a benchmark for the subsequent numerical implementation using a 1-D FDTD scheme as in reference [15], an analytical formulation of the NEAM model with and without thermal and viscous effects has been derived. This linear analytical model provides closed-form expressions for the effective impedance and absorption coefficient, allowing direct comparison with classical impedance-tube theory and with experimental data. Additionally, the development of this analytical model improves the search speed of the NEAM parameters, which can also be used as a starting point in the 1-D FDTD parameter optimization scheme.

Following the NEAM analytical implementations, the robustness of the model was systematically evaluated, understanding that the inherent limitations in experimental data acquisition can introduce uncertainties that affect the estimation of the model’s coefficients. To carry out this study, a sweep close to the optimal value of each coefficient was performed with a margin of ±20%. For calculation simplicity, the linear model without thermo-viscous losses was used.

## 2. Materials and Methods

This section presents the methodological tools employed in this study. In Section 2.1, the NEAM analytical model is described, forming the foundation of the proposed framework. Section 2.2 extends this formulation to include thermo-viscous effects, defining the thermo-viscous NEAM analytical model. In Section 2.3, the thermo-viscous FDTD NEAM model is introduced, which serves as the core of the numerical simulation environment. Finally, Section 2.4 details the adaptive grid technique used to efficiently resolve multi-scale structures while reducing computational cost.

### 2.1. Analytical NEAM Model

For normal incidence, the surface impedance of a thin air layer of thickness d in front of a rigid boundary can be expressed as

(1) $$Z_{p}=-{jZ}_{0}\cdot cot(kd).$$

where $Z_{0}=\rho_{0}c$ is the characteristic impedance of air and k is the acoustic wavenumber. This expression shows that a thin air layer behaves like a spring with compliance $d/\rho_{0}c^{2}$.

To extend this concept to a porous layer, the air impedance $Z_{0}$ can be replaced by the porous characteristic impedance $Z_{cp}$, and the wavenumber k by the complex wavenumber of the porous medium ${\hat{k}}_{p}$, giving

(2) $$Z_{p}=-{jZ}_{cp}\cdot \cot\left({\hat{k}}_{p}d\right).$$

The two unknowns $Z_{cp}$ and ${\hat{k}}_{p}$ are obtained from the NEAM linear model [15], whose constitutive equations for pressure p and particle velocity v are recollected here for convenience

(3) $$\Psi_{A}\frac{\partial p}{\partial t}+\Psi_{B}p=-\rho_{0}c^{2}\nabla v,$$

(4) $$\Omega_{A}\frac{\partial v}{\partial t}+\Omega_{B}v=-\rho_{0}^{-1}\nabla p.$$

where $\Omega_{A},\Omega_{B},\Psi_{A},\Psi_{B}$ are the NEAM linear model parameters, $\rho_{0}$ the static density, and c the sound speed.

In harmonic regime, both pressure and particle velocity can be expressed in exponential form, with complex coefficients $\hat{A}$ and $\hat{B}$, such that

(5) $$\hat{p}=\hat{A}e^{j(\omega t-{\hat{k}}_{p}x)},\;\;\hat{v}=\hat{B}e^{j(\omega t-{\hat{k}}_{p}x)}.$$

Substituting into the constitutive Equations (3) and (4) and collecting spatial terms yields

(6) $$j\Psi_{A}\hat{A}\omega +{\hat{A}\Psi }_{B}=j\rho_{0}c^{2}\hat{B}{\hat{k}}_{p},$$

(7) $$\;j\Omega_{A}\hat{B}\omega +\hat{B}\Omega_{B}=\frac{j\hat{A}{\hat{k}}_{p}}{\rho_{0}}.$$

The characteristic impedance is then defined as the ratio of sound pressure to particle velocity

(8) $$Z_{cp}=\frac{\hat{P}}{\hat{v}}=\frac{\hat{A}e^{j(\omega t-{\hat{k}}_{p}x)}}{\hat{B}e^{j(\omega t-{\hat{k}}_{p}x)}}=\frac{\hat{A}}{\hat{B}}.$$

From Equations (6) and (7), solving for $\hat{A}$ and $\hat{B}$, and inserting in (8) leads to the porous characteristic impedance

(9) $$Z_{cp}=\frac{\hat{P}}{\hat{v}}=\frac{\hat{A}}{\hat{B}}=Z_{0}\sqrt{\frac{\Omega_{A}-j\frac{\Omega_{B}}{\omega }}{\Psi_{A}-j\frac{\Psi_{B}}{\omega }}}.$$

Note that if $\Omega_{A}=\Psi_{A}=1$ and $\Omega_{B}=\Psi_{B}=0$, which is the case of no absorption,$\;Z_{cp}=Z_{0}$ (same medium). In the case of a porous absorber with only drag forces ($\Omega_{A}=\Psi_{A}=1$, $\Omega_{B}>0,\Psi_{B}=0$), previous equation reduces to

(10) $$Z_{cp}=Z_{0}\sqrt{1-(j\Omega_{B})/\omega }.$$

In order to calculate the surface impedance of NEAM porous absorber, the complex wavenumber shall be obtained. Then, the expressions (6) and (7) are used, and directly organized to clear from the equation ${\hat{k}}_{p}$. Since $\hat{A}/\hat{B}=Z_{cp}$, and $Z_{0}=\rho_{0}c$, this becomes, after simplification,

(11) $${\hat{k}}_{p}=\frac{\omega }{c}\sqrt{\Psi_{A}-j\frac{\Psi_{B}}{\omega }}\sqrt{\Omega_{A}-j\frac{\Omega_{B}}{\omega }}.$$

Finally, substituting the characteristic impedance and complex wavenumber into expression (2) gives the analytical solution of the NEAM model surface impedance:

(12) $$Z_{p}=-jZ_{0}\sqrt{\frac{\Omega_{A}-j\frac{\Omega_{B}}{\omega }}{\Psi_{A}-j\frac{\Psi_{B}}{\omega }}}\cdot cot\left(\frac{d\omega }{c}\sqrt{\Psi_{A}-j\frac{\Psi_{B}}{\omega }}\sqrt{\Omega_{A}-j\frac{\Omega_{B}}{\omega }}\right).$$

As in reference [16], the normal absorption coefficient is calculated as $\alpha_{n}=1-{\left|R_{p}\right|}^{2}$, where $R_{p}$ is the NEAM reflection coefficient obtained as the following impedance ratio:

(13) $$R_{p}=\frac{\left(Z_{p}-Z_{0}\right)}{\left(Z_{p}+Z_{0}\right)}.$$

### 2.2. Analytical Thermo-Viscous NEAM Model

To include viscous and thermal effects in porous materials, we extend the NEAM linear model by introducing numerical thermo-viscous coupling coefficients. These coefficients must account for energy dissipation due to viscous and thermal interactions between pressure and particle velocity fields.

Starting from the linearized Navier–Stokes equations, the continuity equation, and Fourier’s law of heat conduction in a quiescent medium [17,18], the governing equations for pressure p and particle velocity v are

(14) $$\frac{\partial p}{\partial t}=-\rho_{0}c^{2}\left(\nabla \cdot v\right)+\left(\frac{\gamma -1}{\rho_{0}C_{p}}\right){\kappa \nabla }^{2}p,$$

(15) $$\rho_{0}\frac{\partial v}{\partial t}=-\nabla p+\left(\frac{1}{3}\mu +\mu_{B}\right)\nabla \left(\nabla \cdot v\right)+\mu \nabla^{2}v.$$

where γ is the ratio of specific heats, $C_{p}$ the specific heat at constant pressure, K the thermal conductivity, and μ, $\mu_{B}$ the shear and bulk viscosities, respectively. These equations represent the standard thermo-viscous acoustic model in air and constitute the baseline for further FDTD extensions [19,20]. To further develop this framework toward more general dissipative description, auxiliary NEAM coefficients $\Omega_{A},\Omega_{B},\Psi_{A},\Psi_{B}$ and the numerical thermo-viscous coefficients $\Psi_{C},\Omega_{C},\Omega_{D}$ are introduced following the approach of Wilson [21,22]. The modified continuity and momentum equations then become

(16) $$\Psi_{A}\frac{\partial p}{\partial t}+\Psi_{B}p=-\rho_{0}c^{2}\left(\nabla \cdot v\right)+{\Psi_{C}\nabla }^{2}p,$$

(17) $$\Omega_{A}\frac{\partial v}{\partial t}+\Omega_{B}v=\rho_{0}^{-1}\left[-\nabla p+\left(\frac{1}{3}\Omega_{C}+\Omega_{D}\right)\nabla \left(\nabla \cdot v\right)+\Omega_{C}\nabla^{2}v\right].$$

Assuming harmonic wave propagation, $p,v\propto e^{j(\omega t-{\hat{k}}_{p}x)}$, the coupled algebraic system is

(18) $$j\Psi_{A}\omega \hat{p}+\Psi_{B}\hat{p}=j\rho_{0}c^{2}{\hat{k}}_{p}\hat{v}-\Psi_{c}{\hat{k}}_{p}^{2}\hat{p},$$

(19) $$j\Omega_{A}\omega \hat{v}+\Omega_{B}\hat{v}=\frac{j{\hat{k}}_{p}}{\rho_{0}}\hat{p}-\frac{{\hat{k}}_{p}^{2}}{\rho_{0}}\left(\frac{4}{3}\Omega_{C}+\Omega_{D}\right)\hat{v}.$$

where $\hat{p}$ and $\hat{v}$ are the harmonic pressure and particle velocity, and ${\hat{k}}_{p}$ denotes the complex wavenumber. Introducing the thermo-viscous characteristic impedance, $Z_{cp}=\hat{p}/\hat{v}$, allows to express it in terms of pressure. Substituting into Equation (18) gives

(20) $$Z_{cp}\left(j\Psi_{A}\omega +\Psi_{B}+\Psi_{c}{\hat{k}}_{p}^{2}\right)=j\rho_{0}c^{2}{\hat{k}}_{p}.$$

Replacing this expression into Equation (19), and dividing by the particle velocity in both sides, the system, organized, reduces to a single expression in ${\hat{k}}_{p}$

(21) $$\rho_{0}{\hat{k}}_{p}^{2}\left(\frac{4}{3}\Omega_{C}+\Omega_{D}\right)-jZ_{cp}\rho_{0}{\hat{k}}_{p}+j\Omega_{A}\omega +\Omega_{B}=0.$$

Eliminating $Z_{cp}$ from the previous equation systematically leads to a quadratic expression in $x={\hat{k}}_{p}^{2}$ in the form of

(22) $$B_{2}x^{2}+B_{1}x+B_{0}=0,$$

where the coefficients are obtained by collecting terms according to powers of ${\hat{k}}_{p}^{2}$ after the substitution. The quadratic coefficient, $B_{2}$, originates from the combination of the pore-frame compressibility term $\Psi_{c}{\hat{k}}_{p}^{2}$ in the pressure equation and the viscous and thermal damping term $\frac{{\hat{k}}_{p}^{2}}{\rho_{0}}\left(\frac{4}{3}\Omega_{C}+\Omega_{D}\right)$ in the velocity equation, representing shear and bulk visco-thermal effects. The linear coefficient, $B_{1}$, collects the terms proportional to ${\hat{k}}_{p}$. The last constant term, $B_{0}$, contains all remaining contributions, including those cross-terms generated by the elimination procedure. However, for computational efficiency and clarity in the implementation, subexpressions $C_{1}$ and $C_{2}$ are defined to collect the dominant linear and quadratic contributions, such that

(23) $$C_{1}=3c_{0}^{2}\rho_{0}+3i\omega \rho_{0}\Psi_{C}\Omega_{A}+3\rho_{0}\Psi_{C}\Omega_{B}+4i\omega \Psi_{A}\Omega_{C}+4\Psi_{B}\Omega_{c}+3i\omega \Psi_{A}\Omega_{D}+3\Psi_{B}\Omega_{D},$$

(24) $$\begin{array}{cc} C_{2}=12\rho_{0}(\omega \Psi_{A} & -i\Psi_{B})\Psi_{C}\left(\omega \Omega_{A}-i\Omega_{B}\right)\left(4\Omega_{C}+3\Omega_{D}\right)\\ & +{\left(3\rho_{0}\left(c_{0}^{2}+\Psi_{C}\left(i\omega \Omega_{A}+\Omega_{B}\right)\right)+\left(i\omega \Psi_{A}+\Psi_{B}\right)\left(4\Omega_{C}+3\Omega_{D}\right)\right)}^{2}. \end{array}$$

Then, the compact quadratic equation is solved for ${\hat{k}}_{p}$, selecting the branch such that $I\left({\hat{k}}_{p}\right)>0$, yielding

(25) $${\hat{k}}_{p}=\frac{1}{\sqrt{2}}\sqrt{-\frac{1}{\Psi_{C}A_{1}}\left(C_{1}-\sqrt{C_{2}}\right)}.$$

where $A_{1}=4\Omega_{C}+3\Omega_{D}$.

Once the complex wavenumber is determined, the thermo-viscous characteristic impedance is obtained as

(26) $$Z_{cp}=\frac{1}{6A_{2}}{\hat{k}}_{p}\left(C_{3}+\sqrt{C_{2}}\right).$$

where

(27) $$C_{3}=3c_{0}^{2}\rho_{0}+3i\omega \rho_{0}\Psi_{C}\Omega_{A}+3\rho_{0}\Psi_{C}\Omega_{B}-4i\omega \Psi_{A}\Omega_{C}-4\Psi_{B}\Omega_{C}-3i\omega \Psi_{A}\Omega_{D}-3\Psi_{B}\Omega_{D},$$

(28) $$A_{2}=\omega \Psi_{A}-i\Psi_{B}.$$

This reformulation is mathematically equivalent to solving the original quadratic formulae, but it avoids explicitly using $B_{0},\;B_{1},\;B_{2}$ coefficients, making the expressions more compact and numerically stable for implementation. Coefficients $C_{1},C_{2},C_{3}$ arise from systematically eliminating the impedance between the coupled pressure and velocity equations and grouping terms according to powers of ${\hat{k}}_{p}^{2}$. The approach ensures that even small thermo-viscous effects are captured correctly. While the main attenuation is dominated by $\Omega_{B}$, the additional thermo-viscous coefficients refine the absorption curve. The quadratic dispersion relation naturally predicts two propagation regimes, and in the absence of thermo-viscous effects, where $\Psi_{C}=\Omega_{C}=\Omega_{D}=0$, the wavenumber and characteristic impedance reduce to the linear relation, reproducing the initial NEAM solution. Finally, to compute the surface impedance and the absorption coefficient, Equations (2) and (13) must be used with the calculated parameters.

### 2.3. Thermo-Viscous FDTD NEAM Model

The analytical thermo-viscous NEAM model developed in the previous section can be integrated into the time domain through a classical FDTD scheme. In this formulation, the generalized pressure and momentum equations are discretized on a uniform full grid, which enables the explicit computation of both pressure and particle velocity fields while preserving the full thermo-viscous behavior. As an example, a one-dimensional finite difference discretization along the x-axis is developed. The pressure update Equation (16) at node i and time step n+1 can be written as

(29) $${\left.p\right|}_{i}^{n+1}=\left(\frac{\frac{\Psi_{A}}{\Delta t}-\frac{\Psi_{B}}{2}}{\frac{\Psi_{A}}{\Delta t}+\frac{\Psi_{B}}{2}}\right){\left.p\right|}_{i}^{n}-\left(\frac{\rho_{0}c^{2}}{\frac{\Psi_{A}}{\Delta t}+\frac{\Psi_{B}}{2}}\right)\left(\nabla \cdot v\right)+\left(\frac{\Psi_{C}}{\frac{\Psi_{A}}{\Delta t}+\frac{\Psi_{B}}{2}}\right)\nabla^{2}p.$$

Meanwhile, the particle velocity update Equation (16) at the staggered node i+1/2 and half-time step n+1/2 is expressed as

(30) $${\left.v\right|}_{i+1/2}^{n+1/2}=\left(\frac{\frac{\Omega_{A}}{\Delta t}-\frac{\Omega_{B}}{2}}{\frac{\Omega_{A}}{\Delta t}+\frac{\Omega_{B}}{2}}\right){\left.v\right|}_{i-1/2}^{n-1/2}-\left(\frac{\frac{1}{\rho_{0}\Delta x}}{\frac{\Omega_{A}}{\Delta t}+\frac{\Omega_{B}}{2}}\right){\left.p\right|}_{i+1}^{n+1}-{\left.p\right|}_{i}^{n+1}+\left(\frac{\frac{\Omega_{C}}{3\rho_{0}}+\frac{\Omega_{D}}{\rho_{0}}}{\frac{\Omega_{A}}{\Delta t}+\frac{\Omega_{B}}{2}}\right)\nabla \left(\nabla \cdot v\right)+\left(\frac{\frac{\Omega_{C}}{\rho_{0}}}{\frac{\Omega_{A}}{\Delta t}+\frac{\Omega_{B}}{2}}\right)\nabla^{2}v.$$

This staggered-grid arrangement achieves second-order accuracy in space and time and ensures numerical stability under the Courant–Friedrichs–Lewy (CFL) condition. The FDTD formulation naturally extends to multi-dimensional domains by applying the same update rules along all spatial directions. Moreover, boundary conditions can be implemented consistently to represent rigid walls, absorbing layers, or periodic structures, ensuring compatibility with the fundamental algorithm architecture. The resulting FDTD framework provides a numerically robust tool for simulating wave propagation in thermo-viscous porous media, offering a direct complement to the NEAM analytical predictions.

### 2.4. Adaptaive Grid Technique

Finite difference methods on uniform grids are simple and widely used. However, when simulating poro-acoustic waves, the mesh requirements become extremely high because of the smallest spatial feature, or the highest frequency in the model, which dictates a globally fine mesh. Adaptative grids address this issue by varying the cell size in space, concentrating resolution where it is needed, and allowing larger spacing elsewhere and were pioneered by Berger [23]. The fundamental idea is to generate a set of nodal coordinates $x_{i}$ and $y_{i}$ such that the local spacings ${\Delta x}_{i}=x_{i+1}-x_{i}$ and ${\Delta y}_{j}=y_{j+1}-y_{j}$ vary smoothly. Smoothness is essential because sudden changes in grid spacing act as internal boundaries and would generate spurious reflections [24]. Fornberg showed that finite difference approximations with regularized varying intervals retain second-order accuracy [25], and later analyses confirmed FDTD stability under appropriate CFL conditions [26]. In practice, a stretching factor (SF) between 1.1 and 1.4 is empirically used for smooth coarse–fine transitions as reported in classical CFD literature. Under this condition, there are no abrupt changes between cells:

(31) $$\frac{{\Delta x}_{i+1}}{{\Delta x}_{i}}\leq SF.$$

Larger changes in spacing require transition regions, where the cell size should be progressively interpolated. If this condition is violated, even if the CFL condition is satisfied, numerical instabilities or spurious reflections may appear at the coarse-fine interface [27]. Similar effects have been reported in other discretization frameworks such as the lattice Boltzmann method [28], which are analogous to what occurs in FDTD.

In this work, a piecewise refinement strategy with graded transitions is adopted, where the computational domain is divided into regions with different levels of resolution and different grid reduction approaches. Hustedt [29] discusses techniques for handling mixed grids that inform considerations of accuracy and stability in multi-resolution contexts; the current hybrid approach, with graded transitions at the edges and uniform fine spacing in the interior, is implemented to ensure smooth grid variation and efficient concentration of resolution.

A uniform coarse mesh is first employed up to a prescribed interface. At this point, the grid is refined within a central region of thickness $L_{f}$, refined by a ratio r, in order to resolve small-scale structures or material heterogeneities. Inside the refined region, a uniform fine mesh is constructed using a linear interpolation of the cell size:

(32) $$\Delta x\left(k\right)=\Delta x_{coarse}+\frac{k}{M}\left(\Delta x_{fine}-\;\Delta x_{coarse}\right),\;\;k=0,1,\ldots ,M.$$

To further smooth the coarse-fine interface, graded transition zones are introduced at the entrance and exit of the refined region. These transitions follow a nonlinear progression law controlled by a curvature parameter β:

(33) $$G=\left\{x_{i},x_{i}+\Delta x_{1},\ldots ,x_{e}\right\},\;\;\Delta x\left(k\right)=\Delta x_{i}+\left(\Delta x_{e}-\Delta x_{i}\right){\left(\frac{k}{N}\right)}^{\beta },\;\;k=0,1,\ldots ,N.$$

where N is the number of transition intervals, $\Delta x_{i}$ and $\Delta x_{e}$ are the initial and final transition step sizes, and β>0 controls the change rate, such as β=1 being a linear transition, β>1 producing a slow–fast progression, and β<1 a fast–slow one. This generalized approach allows smooth compression and expansion of the grid, extending the original linear idea while providing more flexibility for numerical stability.

In two dimensions the same construction is applied independently in x and y. If $x_{i}=x(\xi_{i})$ and $y_{j}=y(\eta_{j})$ are obtained from mappings ξ→x and η→y, the grid becomes a tensor product mesh, where the 2D grid is constructed as the cartesian product of the 1D grids in each direction [30]. Then, local spacings are $\Delta x_{i}=x_{i+1}-x_{i}$ and $\Delta y_{j}=y_{j+1}-y_{j}$ and each cell has an area of ${\Delta x}_{i}\Delta y_{j}$. The refinement may also be anisotropic, with finer resolution in one direction than the other, as illustrated in reference [31].

Once the non-uniform coordinates are defined, finite-difference operators are written with respect to the local spacing. For instance, the discrete derivative of pressure in the x-direction at a velocity node is

(34) $$\partial_{x}{\left.P\right|}_{i+1/2,j}\approx \frac{P_{i+1,j}-P_{i,j}}{\Delta x_{i}},$$

and the divergence at a pressure node is

(35) $$\partial_{x}{\left.u\right|}_{i,j}\approx \frac{u_{i+1/2,j}-u_{i-1/2,j}}{x_{i+1/2}-x_{i-1/2}},\;\;x_{i+1/2}=x_{i}+\frac{\Delta x_{i}}{2}\;.$$

These formulas show how local spacing enters the finite-difference stencil. Higher-order accuracy can be obtained by solving a system of Taylor expansions, yielding coefficients that explicitly depend on $\Delta x_{i-1}$ and $\Delta x_{i}$. The time step in explicit schemes is dictated by the smallest grid cell in the entire mesh, since the CFL condition involves the minimum local spacing on both directions. Following the stability analysis performed in reference [26], the bound can be written as

(36) $$\Delta t\leq \underset{i,j}{\min}\frac{1}{c_{o}\sqrt{1/{\Delta x}_{i}^{2}+1/{\Delta y}_{j}^{2}}}.$$

In practice, a safety factor is often applied, and Taflove and Hagness [32] recommend choosing Δt around half of this theoretical limit to ensure stability. Nevertheless, this bound is often more restrictive than necessary, and an energy analysis or eigenvalue tests can provide tighter limits. The important point is that refinement zones enforce the global Δt, unless local time-stepping or alternating-direction implicit (ADI) subgridding approaches are used [33]. In this work a CFL factor of 95% was chosen for 1-D FDTD simulations with thermo-viscous effects, and 100% was chosen for non-thermo-viscous cases.

At coarse–fine interfaces, consistency requires special care. Mismatched grids can generate artificial reflections unless fluxes are conserved. Conservative interpolation, averaging, or SBP–SAT approaches preserve stability and discrete energy [34]. In poro-acoustic simulations, these techniques are even more important due to impedance mismatches. Thus, the combination of graded-uniform piecewise refinement with careful interface treatment provides a robust framework for multi-scale poro-acoustic simulations, balancing accuracy, stability, and computational cost.

## 3. Results

Here, efforts have focused on improving the macroscopic response of the absorption coefficient under normal incidence. To achieve this objective, modifications were introduced to the initial NEAM model, resulting in the enhanced version referred to as eNEAM. This section presents the outcomes of analyses performed using the eNEAM framework. All simulations and theoretical calculations were carried out on a 60 mm thick melamine foam, with experimental values obtained from [35]. FDTD simulations, with the source code generated by the authors, were handled with Matlab in a six core AMD Ryzen 5 4500U processor at 3.40 GHz and 32 GB DDR4 RAM with 20 points per wavelength (PPW), considering an upper frequency of $4k\sqrt{2}$. Section 3.1 presents the numerical refinements and parameter contributions in the NEAM model, emphasizing their impact on low-frequency absorption predictions. Section 3.2 examines the robustness of the eNEAM parameters, evaluating the model sensitivity to variations in its coefficient values. Section 3.3 quantifies the adaptive grid technique impact on computational efficiency. Finally, Section 3.4 compares the eNEAM linear model with the thermo-viscous formulation, pointing out the marginal effect of thermo-viscous effects on FDTD predictions.

### 3.1. NEAM Model Enhancements

**Figure 1:**
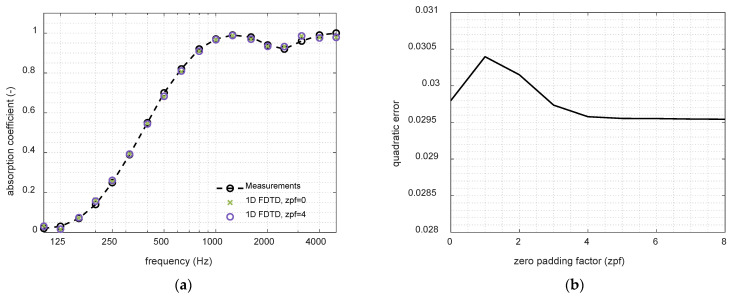
Effect of zero padding in the Fourier Transform of the eNEAM impulse response carried out on a 60 mm thick melamine foam using 1D-FDTD simulations. (**a**) Absorption coefficient results with two different zero padding factors (zpf); (**b**) Errors obtained with different zero padding factors.

The first study was conducted to analyze the influence of the Fourier transform zero padding of the impulse response obtained from FDTD simulations. This point in the NEAM scheme is essential for accurately determining the incident and reflected acoustic energies, from which the reflection and combined transmission–absorption coefficients are subsequently derived. The objective was to evaluate whether increasing the oversampling factor improves the accuracy and resolution of the absorption coefficient spectra. Results show that, while oversampling reduces the error committed, as illustrated in Figure 1, the overall absorption trend remains essentially unchanged, confirming that the eNEAM predictions are not significantly affected by this numerical aspect. Since the use of zero padding decreases the cost-function error and has a negligible impact on computational cost, a factor of 4 is applied in all subsequent analyses.

**Figure 2:**
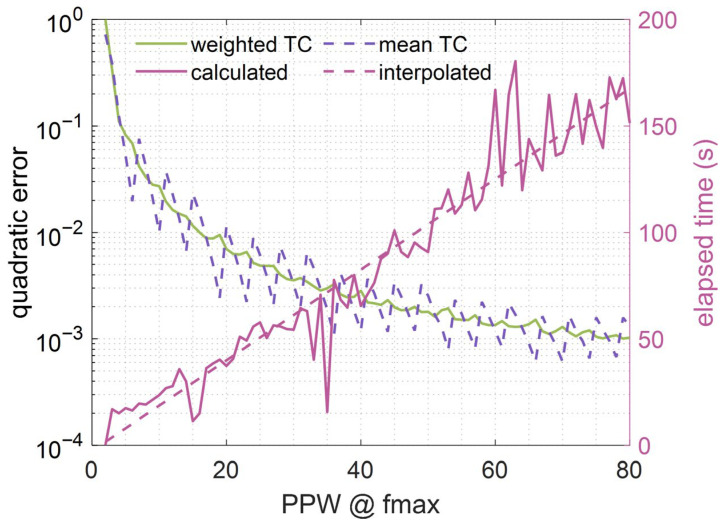
Influence of the transition cell (TC) at the air-porous interface in 1D-FDTD simulations. Figure has two Y-axis. On the left, the algorithm quadratic error used for weighted TCs (green line) and arithmetic mean TCs (purple dashed line). On the right, the computational time required for the simulation, showing both the actual calculated time (magenta line) and its linear regression (magenta dashed line).

Secondly, in the FDTD implementation, a common staggered mesh is employed between particle velocity and pressure. This configuration requires special attention at the transition cell(s) located at the air–porous interface. Conventionally, an arithmetic mean of the velocity coefficients is applied at this interface. However, the analysis shows that this approach is only valid when the real-to-numerical spatial mapping exactly corresponds to an integer, a condition rarely met in practice. As a result, the arithmetic mean introduces imbalances in the interface coefficients and produces abrupt, sawtooth-like variations in the mean square error of the cost function. To overcome this limitation, the coefficients at the interface were weighted, assigning greater weight to one of the two media according to the non-integer portion of the real-to-numerical spatial mapping. As shown in Figure 2, this strategy smooths the error curve and improves the solution stability. Furthermore, Figure 2 also illustrates the PPW required in the FDTD implementation to achieve results comparable to those analytically obtained. Results indicate that using PPW in a 16 to 20 range provides low error levels while maintaining a reasonable computational cost. For better performance, all FDTD simulations carried on in this paper use 20 PPW.

**Figure 3:**
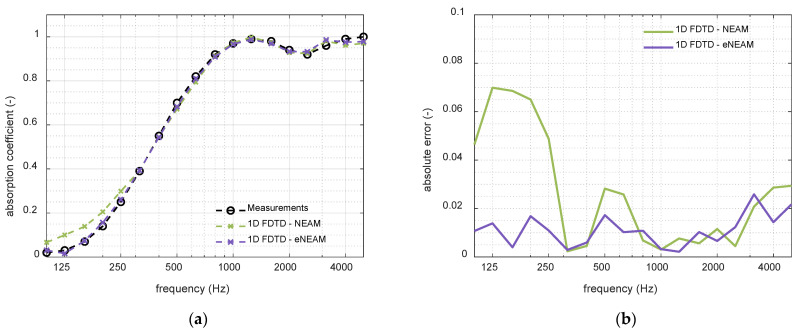
Simulations comparison between NEAM (green) and eNEAM (purple) schemas using the same FDTD configuration and calculated for a 60 mm-thick melamine foam: (**a**) Absorption coefficient results; (**b**) absolute error calculated with measured values as reference.

**Figure 4:**
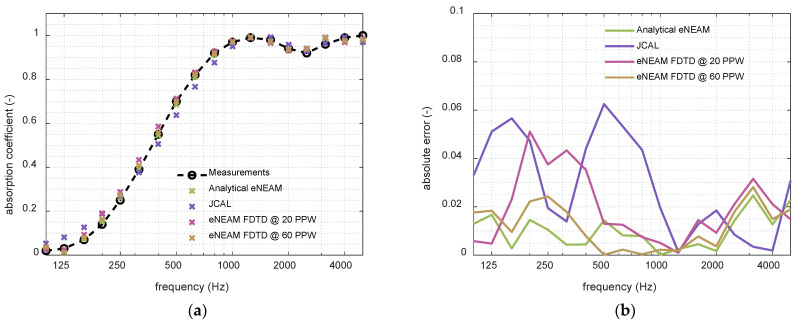
Simulations comparison between analytical eNEAM (green), JCAL model (purple), and numerical eNEAM at 20 points per wavelength (magenta) and numerical eNEAM at 60 points per wavelength (brown), calculated for a 60 mm-thick melamine foam: (**a**) Absorption coefficient results; (**b**) absolute error calculated with measured values as reference.

Finally, the last step of the study was to individually analyze the NEAM parameters’ contribution to the overall absorption response, revealing that allowing the thermolability coefficient, $\Psi_{B}$, to take negative values significantly enhances the model’s accuracy, mainly at low frequencies. Physically, $\Psi_{B}$ quantifies the thermal exchange between the fluid and the solid frame that modulates the material’s effective properties. Rapid pressure fluctuations within the porous material generate compressions and rarefactions, which produce abrupt temperature variations. Compressions correspond to temperature increases (positive $\Psi_{B}$), while rarefactions correspond to temperature decreases (negative $\Psi_{B}$). At low frequencies, the oscillations of the wave are slower, which allows the thermal boundary layer more time to exchange heat between the fluid and the solid frame. This extended interaction time enhances the conversion of acoustic energy into heat, making thermal energy loss more pronounced at low frequencies. Figure 3 shows the absorption coefficient obtained from the 1-D FDTD scheme for NEAM and eNEAM compared to the impedance-tube measurements. It can be observed that in the low frequencies below 315 Hz, the eNEAM method improves the overall absorption response. Also, a slight upturn is observed in the mid-range over 500 Hz. In addition, a better agreement is equally noticeable at higher frequencies (4–5 kHz). This improvement was also incorporated into the analytical model, as shown in Figure 4, where eNEAM methodology is compared to the JCAL model (JCAL parameter values were obtained from [35]). Figure 4 demonstrates minimal differences between eNEAM predictions and measurements in both analytical and FDTD simulations, confirming the overall accuracy and performance of the eNEAM model.

### 3.2. eNEAM Parameters Robustness

**Figure 5:**
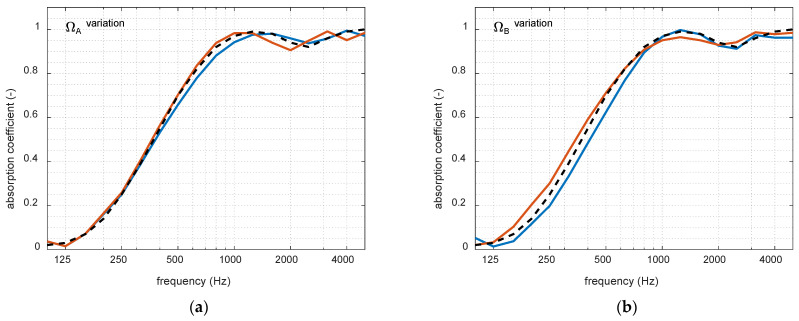

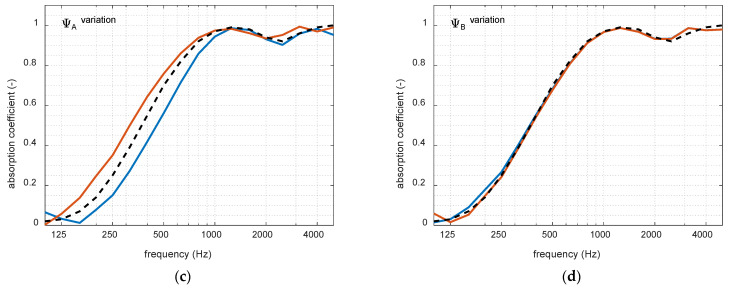
Individual effect of each eNEAM parameter variation analyzed using 1D-FDTD simulations calculated for a 60 mm-thick melamine foam. Each variation only affects one parameter at a time in a −20% (blue lines) to +20% (red lines) range: (**a**) numerical tortuosity ($\Omega_{A}$); (**b**) numerical viscosity ($\Omega_{B}$); (**c**) numerical compressibility ($\Psi_{A}$); (**d**) numerical thermolabile ($\Psi_{B}$).

**Figure 6:**
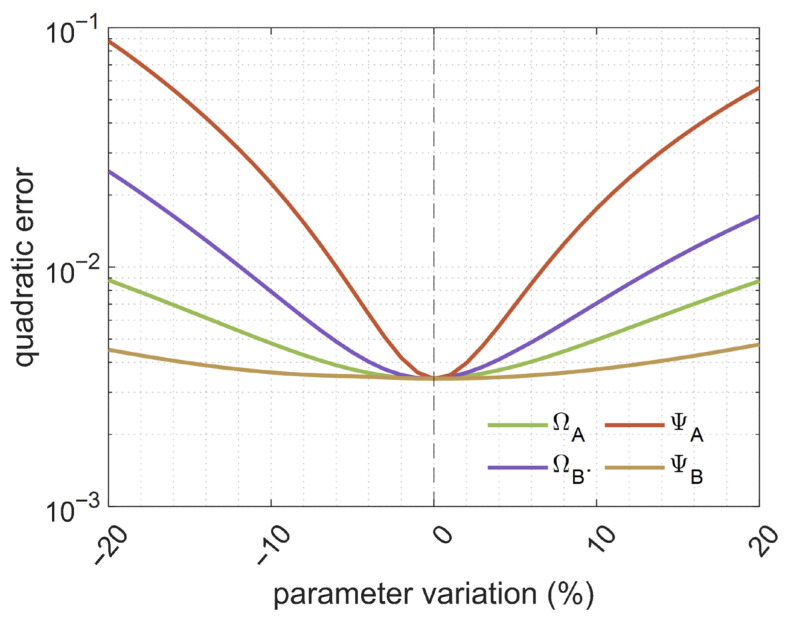
Quadratic error obtained from 1D-FDTD simulations conducted for the robustness study, considering eNEAM parameters variation in the −20% to +20% range calculated for a 60 mm-thick melamine foam.

As discussed throughout this document, the eNEAM model relies on experimental data, which may not always be obtained with high precision. Variations in these measurements can directly affect the model parameters, potentially leading to significant deviations. To address this issue, a robustness study was conducted in which each parameter was individually varied within a ±20% range around its optimally calculated value. Figure 5 shows the individual effect of these variations on each parameter using FDTD simulations, where the blue line represents the lower bound (−20%), the red line represents the upper bound (+20%), and the black dashed line indicates the optimum simulation. It can be observed that only the numerical compressibility parameter ($\Psi_{A}$) has minimal effect on the overall absorption behavior. Finally, Figure 6 summarizes the eNEAM parameters robustness, also revealing an asymmetric behavior, with overestimations having a smaller impact than underestimations. Therefore, results of this analysis demonstrate that, despite these variations, the eNEAM model maintains stable and accurate predictions, confirming its reliability for practical applications within an approximate ± 10% range, resulting in minimal error.

### 3.3. Computational Efficiency Trhough Adaptive Grid

**Figure 7:**
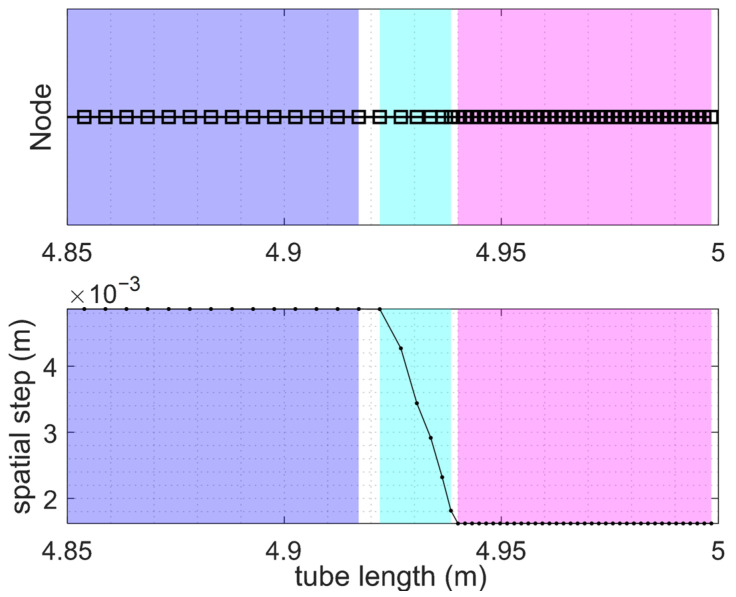
1D-FDTD adaptive mesh configuration showing node accumulation (**top**) and the corresponding FDTD time step (**bottom**). The plot highlights the coarse region (purple), the coarse–fine transition (cyan), and the fine region (magenta).

**Figure 8:**
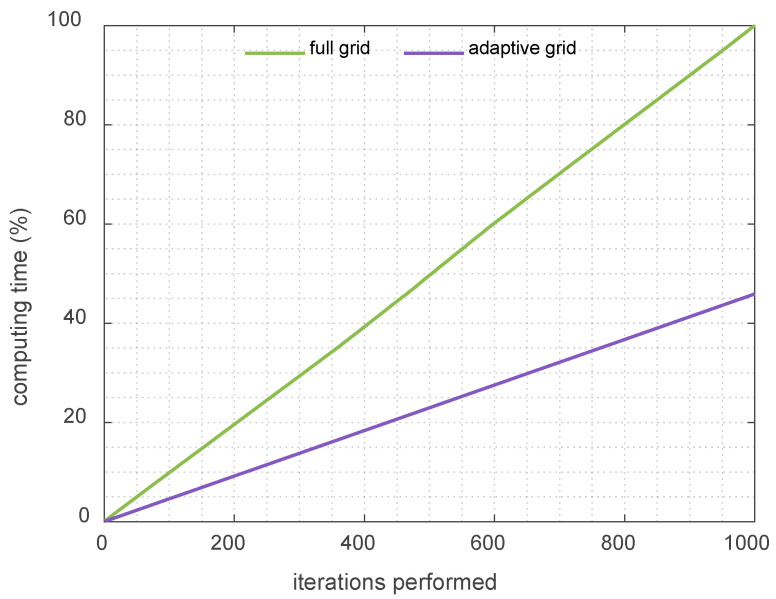
Computation time analysis performed using 1D-FDTD with full grid (green line) compared to the analogous adaptive grid (purple line). Results show the normalized computed time, as a percentage, with the full grid as reference.

**Figure 9:**
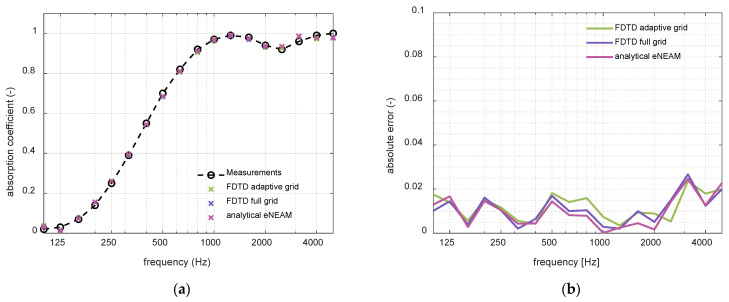
eNEAM model comparison between FDTD simulations with adaptive grid (green), FDTD with full grid (purple), and analytical calculation (magenta): (**a**) absorption coefficient results; (**b**) absolute error with real measurements as reference.

The FDTD-eNEAM computational efficiency was evaluated using a piecewise linear adaptive grid as described in previous sections. In this approach, the computational domain is divided into regions with different resolutions, employing linear refinement within the central high-resolution zones while keeping coarser spacing elsewhere. In the porous region, a target resolution of 30 PPW was employed to accurately resolve small-scale features and material heterogeneities while using a refinement ratio of 3 between the coarse and fine regions. This strategy concentrates grid points in regions of high spatial gradients, such as thin boundary layers or material interfaces, while minimizing the total number of cells. Compared to a uniform mesh with equivalent fine resolution, the adaptive grid reduces the total number of cells and the computational cost of each iteration by approximately 50%. Consequently, the computational cost of the whole project is also reduced by the same amount. Figure 7 and Figure 8 illustrate the mesh configuration and the corresponding reduction in computational time, respectively. The accuracy of the adaptive grid was verified against the analytical eNEAM solution, confirming that stability and low dispersion are preserved as shown in Figure 9.

**Figure 10:**
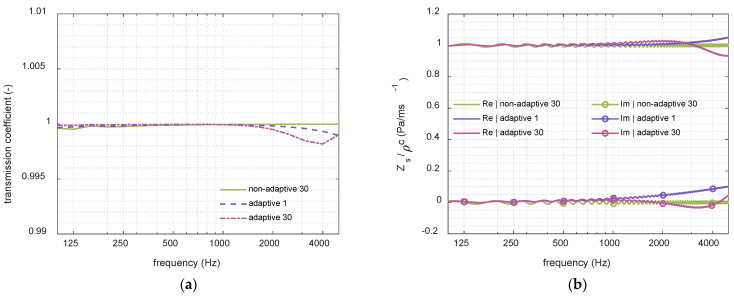
1D-FDTD transmission results of an AIR–eNEAM–AIR interlayer without dissipative effects in the eNEAM model using different numbers of domain cells. The figure shows the non-adaptive grid with 30 cells (green), the adaptive grid with 1 cell (purple), and the adaptive grid with 30 cells (magenta) for (**a**) transmission coefficient and (**b**) surface impedance.

Continuing with the analysis of adaptive dispersion effects on the simulation results, a simplified FDTD configuration was implemented in which an inert eNEAM layer was placed at the center of the tube to examine the influence of different spatial discretization. In this setup, a multilayer AIR–eNEAM–AIR system was constructed, with perfectly matched layers applied at both tube ends. The objective of this simulation was to compute the transmission coefficient using different numbers of fine cells and to compare the results with those obtained from the original, non-adaptive, eNEAM–FDTD formulation. In the non-adaptive configuration, 30 uniform cells were used, whereas the adaptive grid employed regions with 1 and 30 fine cells, respectively. The eNEAM coefficients were selected such that no absorption was introduced into the system, allowing the transition cells and fine–coarse interfaces to be preserved without any dissipative effects. Theoretically, the reflection coefficient of this system should be zero, and the transmission coefficient should be unity. As shown in Figure 10a, the simulated transmission coefficient remains unchanged, exhibiting only negligible deviations at high frequencies. Furthermore, the computed surface impedance demonstrates that the use of an adaptive grid has a minimal impact, with the observed ripple being primarily attributed to the intrinsic numerical dispersion of the FDTD scheme, as depicted in Figure 10b. These results demonstrate that the combination of linear fine regions with curvature-controlled transitions provides a robust framework for multi-scale poro-acoustic simulations, efficiently balancing accuracy, stability, and computational cost without the use of GPU acceleration.

### 3.4. Linear Versus Thermo-Viscous FDTD eNEAM Model

**Figure 11:**
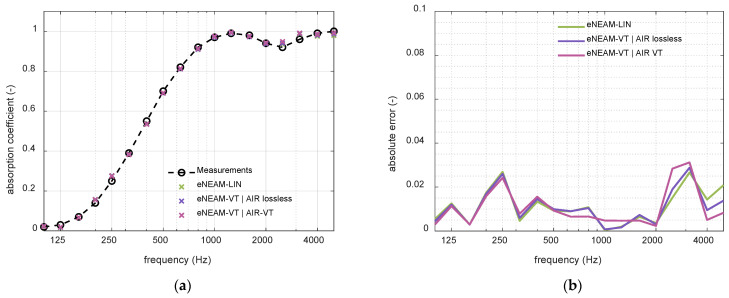
1D-FDTD absorption results deviation, for a 60 mm-thick melamine foam, using the linear eNEAM model (green), the visco-thermal eNEAM without air losses (purple), and the visco-thermal eNEAM including visco-thermal air losses (magenta) for (**a**) absorption coefficient and (**b**) absolute error with real measurements as reference.

**Figure 12:**
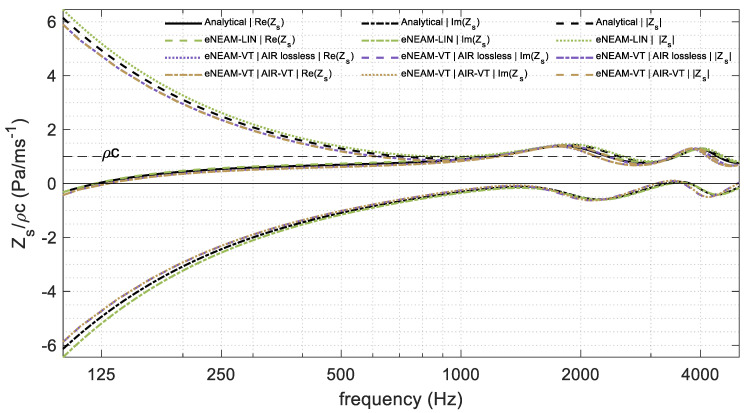
1D-FDTD surface impedance results comparison, for a 60 mm-thick melamine foam, using the analytical eNEAM model (black), the linear eNEAM model (green), the visco-thermal eNEAM without air losses (purple), and the visco-thermal eNEAM including visco-thermal air losses (brown).

The numerical simulations carried out with both the linear and the thermo-viscous FDTD-eNEAM formulations show that the differences between the two approaches are negligible within the whole frequency range considered in this study. The inclusion of thermo-viscous terms slightly modifies the normal absorption prediction, but their contribution remains minor compared to the dominant dissipative mechanism already represented by the drag-related coefficients of the linear eNEAM model. As a result, the predicted absorption coefficient and the surface impedance obtained with the thermo-viscous scheme almost overlap with those calculated using the analytic and FDTD linear formulation as shown in Figure 11 and Figure 12.

In addition to the small effect on the overall acoustic performance, the thermo-viscous formulation also leads to an increased computational cost due to the higher number of operations required in each update step and the use of extra matrices for the new coefficients. This additional load is not justified for the cases under consideration, where the linear FDTD-eNEAM model already provides accurate results with lower memory and time requirements. Therefore, the linear formulation constitutes a more efficient choice for most engineering applications involving porous layers of moderate flow resistivity and typical audible excitation frequencies. The thermo-viscous extension may only become relevant in extreme cases, such as very fine microstructures, very high frequencies, non-linearities, or when precise modeling of secondary attenuation effects is required.

## 4. Conclusions

This work has presented the Enhanced Numerical Equivalent Acoustic Material (eNEAM) framework as an extension of the original NEAM formulation, combining analytical derivations, parameter optimization, and time-domain numerical simulations. The analytical model provided closed-form expressions for surface impedance, complex wavenumber, and absorption coefficient, both with and without thermo-viscous effects, allowing direct validation against impedance-tube measurements and serving as a foundation for numerical implementation. The introduction of negative values of the thermolability coefficient ($\Psi_{B}$) proved particularly effectiveness, especially at the low-frequency absorption prediction, thereby reducing systematic discrepancies between simulations and experiments. A robustness analysis further confirmed that the eNEAM model remains stable even when material parameters vary by as much as ± 10%, demonstrating its resilience to uncertainties, typically encountered in experimental characterization. The comparison between the linear and thermo-viscous formulations revealed that the inclusion of thermo-viscous terms does not lead to significant improvements in predictive capability for the audible frequency ranges, while it considerably increases computational cost. For this reason, the linear eNEAM formulation can be considered as a more efficient and practical choice in most engineering applications, with the thermo-viscous extension only becoming relevant under extreme conditions. In terms of numerical efficiency, the adaptive finite-difference time-domain implementation showed that simulation time could be reduced by around 50% in one-dimensional cases, even without GPU acceleration, while preserving accuracy and stability.

Overall, the results demonstrate that eNEAM constitutes a versatile, accurate, and computationally efficient framework for porous media modeling by bridging experimental characterization, analytical formulations, and advanced numerical schemes. The proposed methodology establishes itself as a robust alternative to classical semi-phenomenological models and offers a promising tool for applications in noise control and the design of complex acoustic metamaterials.

## Data Availability

The original contributions presented in the study are included in the article, further inquiries can be directed to the corresponding author.

## Abbreviations

The following abbreviations are used in this manuscript:

JCAL	Johnson–Champoux–Allard–Lafarge
NEAM	Numerical Equivalent Acoustic Material
eNEAM	Enhanced Numerical Equivalent Acoustic Material
FDTD	Finite Difference Time Domain
TPMS	Triply Periodic Minimal Surface
CFL	Courant–Friedrichs–Lewy
CFD	Computational Fluid Dynamics
GF	Grow Factor
ADI	Alternating-Direction Implicit
PPW	Points Per Wavelength
